# Supplementary material for: A Biallelic Truncating Variant in the TPR Domain of GEMIN5 Associated with Intellectual Disability and Cerebral Atrophy
Source: Genes (Basel). 2023 Mar 13;14(3):707. doi: 10.3390/genes14030707 (PMC10048441; doi:10.3390/genes14030707)
Supplement: Supplementary file 1 [file genes-14-00707-s001.zip › Table S1.pdf]

Supplementary Table 1: Homozygous variants shared by II.3 and II.4 siblings

Chrom	Start	Ref	Alt	Gene_symbol	CADD_score_scaled	HGVS	GERP_score	Polyphen_pred (Score)	Sift_pred (Score)	GnomAD	
chr5	154278181	GCAT	G	*GEMIN5*	/	NM_015465.5:c.3162_3164del; (p.Asp1054_Ala1055delinsGlu)	5.76	/	/	/	OMIM #619333
chr8	62412063	G	A	*CLVS1*	21.8	NM_173519.3 ,c.190>A (p.Glu64Lys)	5.67	possibly_damaging -0.64	deleterious_low_confidence (0.01)	/	Homozygous missense variant associated with a nephrotic syndrome *(Lane et al 2022)*
chr11	102646041	C	T	*MMP10*	15.15	NM_002425.3 ,c.944C>A (p.Arg315Gln)	3.27	probably_damaging (0.993)	deleterious (0.03)	3hom	Homozygous in gnomAD
chr17	18063314	C	A	*MYO15A*	14.76	NM_016239.4, c.231C>A (p.Asp77Glu)	3.53	unknown (0.0)	tolerated (0.08)	/	Deafness (OMIM: #600316)
chr11	113619007	T	C	*ZW10*	13.69	NM_004724.4 ,c.1061T>G (p.Asn354Ser)	3.46	benign (0.015)	tolerated (0.14)	3hom	Homozygous in gnomAD
chr17	37866651	C	T	*ERBB2*	13.01	NM_001289938.2 (p.Thr243Ile)	2.41	benign (0.002)	tolerated (0.98)	/	Low prediction scores
chr17	37824780	C	A	*PNMT*	9.43	NM_002686.4 (p.Pro18Thr)	0.63	benign (0.005)	tolerated (0.2)	/	Low prediction scores
chr19	55255448	C	A	*KIR2DL3*	6.87	NM_015868.3 ,c.576G>A (p.His192Gln)	-1.45	benign (0.008)	tolerated (0.14)	/	Low prediction scores
chr2	114257555	T	C	*FOXD4L1*	6.7	NM_012184.5, c.722G>C (p.Leu241Pro)	2.56	benign (0.0)	tolerated (1.0)	960hom	Homozygous in gnomAD
chr16	57092050	G	T	*NLRC5*	5.87	NM_001384972.1 , c.2248G>T (p.Gly750Cys)	3.73	probably_damaging (0.984)	tolerated (0.56)	/	Low prediction scores
chr17	37985633	G	A	*IKZF3*	5.61	NM_001284514.2,c.163+7G>T	-0.32	spliceAI=0	MaxEntScanDiff=/	/	Low prediction scores
chr1	33430102	T	G	*RNF19B*	5.37	NM_153341.4, c.185T>C (p.Gln62Pro)	0.3	unknown(0.0)	tolerated_low_confidence (0.28)	12057homo	Homozygous in gnomAD
chr2	113482965	C	T	*NT5DC4*	4.36	NM_001393655.1,c.893G>T (p.Ser298Leu)	1.62	benign (0.15)	tolerated (0.1)	/	Low prediction scores

chr17	17062002	C	T	*MPRIP*	1.42	NM_015134.4 (p.Pro578Ser)	0.73	benign (0.001)	tolerated (0.47)	5Homo	Homozygous in gnomAD
chr2	114257369	C	A	*FOXD4L1*	0.1	NM_012184.5 (p.Thr179Asn)	1.34	benign (0.002)	tolerated (1.0)	136hom	Homozygous in gnomAD
chr22	21902082	A	G	*RIMBP3C*	0.03	NM_001128633.2,c.2902T>C(p.Trp968Arg)	-2.96	benign (0.43)	tolerated (0.28)	/	Low prediction scores
