# Supplementary figures and images for: A Biallelic Truncating Variant in the TPR Domain of GEMIN5 Associated with Intellectual Disability and Cerebral Atrophy
Source: Genes (Basel). 2023 Mar 13;14(3):707. doi: 10.3390/genes14030707 (PMC10048441; doi:10.3390/genes14030707)

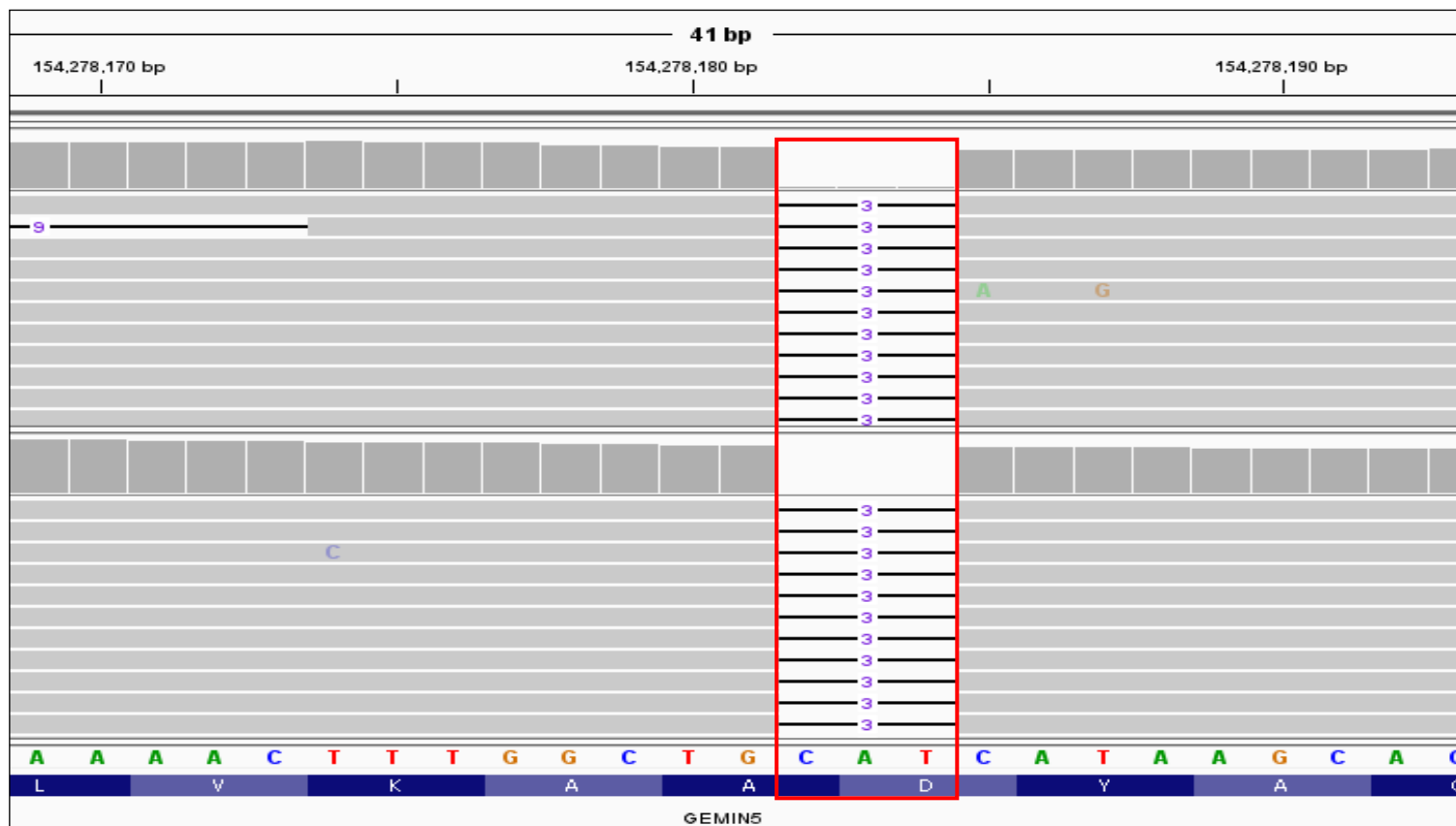

(a)

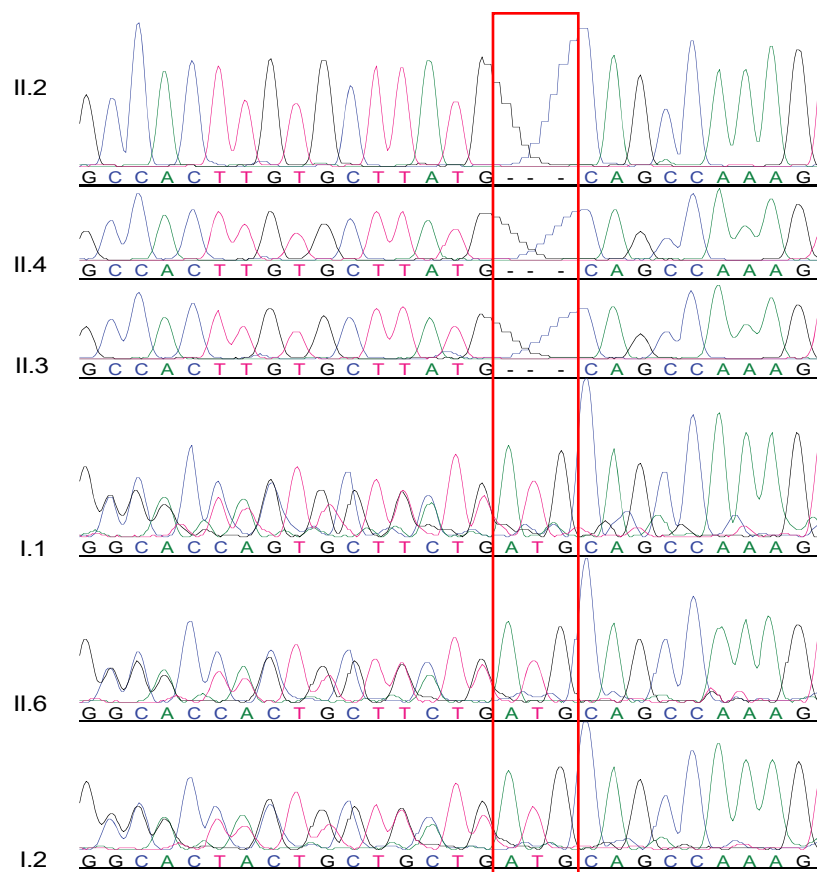

(b)

Supplement: Supplementary file 1 [file genes-14-00707-s001.zip › Figure S1.pdf]

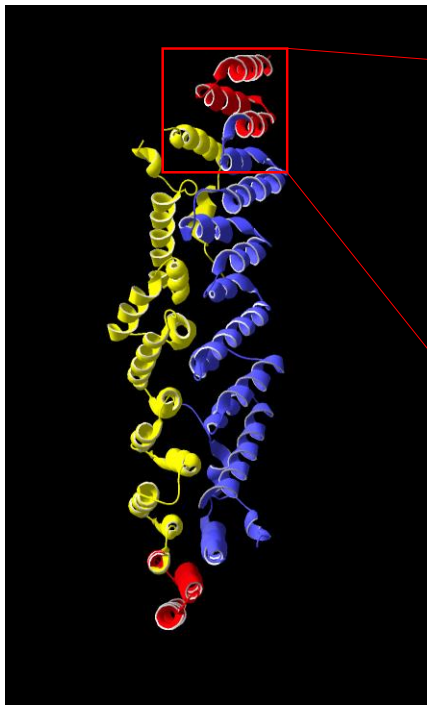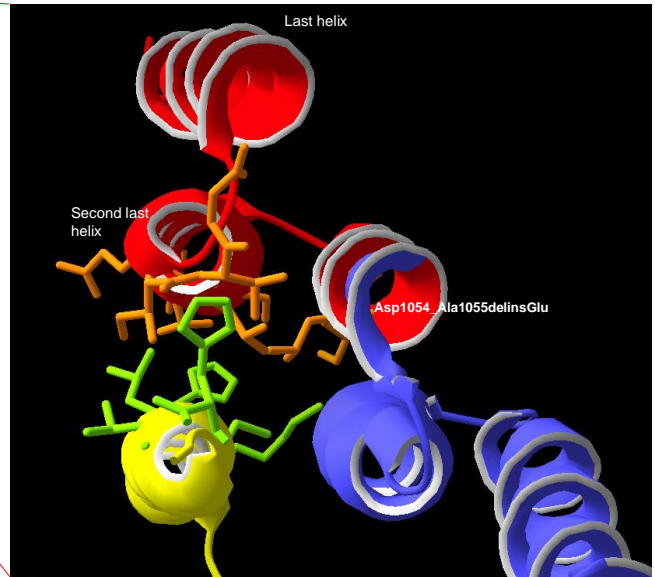

Supplement: Supplementary file 1 [file genes-14-00707-s001.zip › Figure S2.pdf]
